# Behandlungserwartungen und Schmerz

**DOI:** 10.1007/s00482-022-00649-7

**Published:** 2022-05-31

**Authors:** Ulrike Bingel, Regine Klinger

**Affiliations:** 1 Klinik für Neurologie, Universitätsmedizin Essen, Hufelandstr. 55, 45147 Essen, Deutschland; 2 Zentrum für Anästhesiologie und Intensivmedizin, Klinik und Poliklinik für Anästhesiologie, Universitätsklinikum Hamburg-Eppendorf (UKE), Martinistr. 52, 20246 Hamburg, Deutschland

Ein Zuckerdragee vertreibt die Kopfschmerzen, eine Infusion mit Kochsalzlösung lindert die Rückenschmerzen, die Terminbestätigung des monatelang ersehnten schmerztherapeutischen Erstkontakts hebt die Stimmung – kann das möglich sein? Ja, es kann möglich sein! Die Beispiele haben einen gemeinsamen Nenner, es geht um die positive Erwartung einer erfolgreichen Schmerzbehandlung. Allein diese positive Erwartung kann das Schmerzerleben lindern. Denn Erwartungen sind die treibende Kraft von sog. Placeboeffekten. Diese sind seit der Antike beschrieben. Alle Behandelnden beobachten sie in der täglichen Praxis. Auch in klinischen Studien treten hohe Placeboraten auf, die eine große und sogar zunehmende Herausforderung für die Entwicklung und Zulassung neuer Medikamente darstellen.

Die Placeboanalgesie ist von allen in der Literatur beschriebenen Placeboeffekten (Finniss et al. [1]) der bislang am besten untersuchte Placeboeffekt. Die Grundlagen und Mechanismen des analgetischen Placeboeffekts sind vergleichsweise gut verstanden. Lernen, Beobachtung und (verbale) Informationen bilden Erwartungen aus und führen zu Placeboeffekten. Die Anfänge der Entschlüsselung der ihm zugrunde liegenden Mechanismen reichen über 50 Jahre zurück. Damals entdeckten die Pioniere Levine, Gordon und Fields (1979), dass die Placeboanalgesie mit der Ausschüttung von endogenen Opioiden einhergeht [2]. Dabei wurde deutlich: Placeboeffekte sind weder eingebildet noch lästige Artefakte in klinischen Studien, sondern sehr konkrete psychoneurobiologische Vorgänge.

Eine weitere wichtige Erkenntnis: Das Auftreten von positiven Erwartungen an eine Behandlung und gefolgt von Placeboeffekten ist weder an die Behandlung mit Scheinmedikamenten noch an die historisch oft damit verbundene Täuschung des Patienten geknüpft. Jede medizinische Behandlung findet im Kontext individueller Erwartungen und Vorerfahrungen statt. Diese beeinflussen nicht nur die Wirkung von Placebos, sondern auch die von etablierten, evidenzbasierten Behandlungen. Positive Erwartungen können die Wirksamkeit von Analgetika steigern und andere schmerztherapeutische Interventionen stark beeinflussen. Patient:innen mit Schmerzen haben hohe Erwartungen an Schmerzbehandlungen. Werden diese erfüllt, dann treten infolge dieser Erwartungen große Placeboeffekte auf. Die Placeboeffekte „satteln“ sich auf die Effekte der wirksamen Behandlung auf, sodass diese sogar optimiert wird.

Patient:innen mit Schmerzen haben hohe Erwartungen an Schmerzbehandlungen

Die hieran beteiligten Lernmechanismen können auch gänzlich unbewusst stattfinden und trotzdem eine große Wirkung entfalten. Leider nicht immer eine positive: Negative Erwartungen durch negative Erfahrungen, Ängste und Sorgen können Schmerzen verstärken, neue Symptome auftreten lassen oder diese verschlimmern. Diese negativen Folgen einer ungünstigen Erwartung werden auch als Noceboeffekte bezeichnet und sind im klinischen Alltag vermutlich für einen Großteil unerwünschter Medikamentenwirkungen verantwortlich und können schwerwiegende Folgen für die Therapietreue haben. Noceboeffekte vermögen die Wirksamkeit von Analgetika zu reduzieren oder sogar ganz aufzuheben und sind die Ursache, dass Behandlungen weit hinter ihren möglichen Erfolgen zurückbleiben. Wie Noceboeffekte auch das Absetzen von Medikamenten, wie bspw. Antidepressiva, erschweren, ist Gegenstand aktueller Untersuchungen.

Was wirkt, ist also nicht das Placebo selbst, sondern die daran geknüpfte Erwartung. Während die psychologischen Wirkvariablen von Placebo- und Noceboeffekten bereits recht gut verstanden werden, gibt es bzgl. der neurobiologischen und physiologischen Mechanismen von Placeboeffekten und deren Wechselwirkung mit aktiven pharmakologischen Substanzen noch viel zu erforschen. Erfreulicherweise widmet sich der neu eingerichtete DFG-Transregio-Sonderforschungsbereich 289 „Treatment Expectation“ mit einem interdisziplinären Team von Wissenschaftler:innen dieser Thematik und lässt spannende neue Erkenntnisse erwarten.

Die bereits gewonnenen Erkenntnisse können und sollten wir schon jetzt nutzen. Hier ist die Schmerztherapie, bei der die systematische Nutzung von Placeboeffekten und die Vermeidung von Noceboeffekten sogar in der AWMF-Leitlinie „Behandlung akuter perioperativer und posttraumatischer Schmerzen (Registernummer 001-025)“ verankert ist, ein Vorreiter.

Und das können wir auch: Die Behandlungserwartungen und -erfahrungen unserer Patient:innen sind nicht in Stein gemeißelt, sondern werden ganz wesentlich von uns mit beeinflusst. Unsere Kommunikationskompetenz und die Behandler-Patienten-Beziehung sind das derzeit beste Instrument, um die komplexen psychoneurobiologischen Prozesse von Placebo- und Noceboeffekten systematisch zum Wohle des Patienten zu nutzen. Das ist kein Hokuspokus, sondern wissenschaftlich gut fundiert.

In dem aktuellen Themenheft beleuchten renommierte Wissenschaftler:innen das Phänomen von verschiedenen Seiten, dokumentieren den aktuellen Erkenntnisstand und leuchten Zukunftsfelder aus. Dazu gehören die spannenden Fragen:

- Wie wirken Behandlungserwartungen bei akuten Schmerzen, z. B. nach Operationen?
- Welche Rolle spielen Erwartungen bei entzündungsassoziierten Symptomen?
- Welchen Einfluss hat die Therapieerwartung bei der multimodalen Therapie von chronischen Schmerzerkrankungen?
- Welche Rolle spielen negative Erwartungen bei Viszeralschmerzen?
- Können Behandlungserwartungen Hautschmerz und Juckreiz erzeugen?
- Wie können wir das soziale oder Beobachtungslernen zur Optimierung von Placeboeffekten nutzen?
- Warum wirken Placebos sogar, obwohl die Patient:innen wissen, dass sie eine Tablette ohne Wirkstoff erhalten?

Erfahrenen Behandler:innen ist bewusst, wie hilfreich die richtige Kommunikation zwischen Therapeut:innen und Patient:innen ist und wie schädlich eine schlechte sein kann. Die Erforschung des Zusammenhangs von Erwartung der Patient:innen und dem Heilerfolg bietet ein weiteres – evidenzbasiertes – Argument, diese Kommunikation auf allen Ebenen zu fördern: in der Ausbildung, in der täglichen Praxis und nicht zuletzt im Vergütungssystem.

Ihre

Ulrike Bingel & Regine Klinger
